# Research note: Application and validation of RP-HPLC for quantifying ovomucoid, lysozyme, ovotransferrin, and ovalbumin in commercial pasteurized egg white

**DOI:** 10.1016/j.psj.2026.106519

**Published:** 2026-01-25

**Authors:** Ingrid Sousa, Elena Visentin, Silvia Sabbadin, Marta Pozza, Marco Birolo, Massimo De Marchi, Giovanni Niero

**Affiliations:** Department of Agronomy, Food, Natural resources, Animals and Environment, University of Padova, Viale dell’Università 16, Legnaro, (PD) 35020, Italy

**Keywords:** Egg white, Protein composition, RP-HPLC, Method validation, Pasteurization effect

## Abstract

Pasteurized egg white is widely used in the food industry for its high microbiological safety, ease of handling, and versatile technological properties. However, heat-induced protein denaturation during pasteurization can affect its foaming, gelling, and emulsifying functionality. The present study aimed to validate a reversed-phase high-pressure liquid chromatography method for the simultaneous quantification of four major egg white proteins in commercial pasteurized samples, including ovomucoid, lysozyme, ovotransferrin, and ovalbumin. Ten cartons of commercial pasteurized egg white from different brands underwent chromatographic testing, with multiple aliquots analyzed over five consecutive days. The method demonstrated excellent repeatability and reproducibility across all proteins, with ovotransferrin and ovalbumin showing the best performances. Recovery rates ranged from 90.67% for lysozyme to 114.10% for ovomucoid, both at the medium spiking level. Method linearity was assessed using ten serial dilutions of pasteurized egg white in water (1:20 to 1:100). Linear regression of peak areas versus nominal protein concentrations yielded correlation coefficients above 0.99 for all target proteins, confirming a strong proportional response. Concentration of ovomucoid, lysozyme, ovotransferrin, and ovalbumin averaged 15.23, 2.23, 13.50, and 71.80 mg/mL (respectively), which suggests minimal impact of pasteurization on the egg white protein composition. The chromatographic method validated in the present study provides a reliable and practical tool for both research and industrial applications, enabling accurate monitoring of protein composition in commercial pasteurized egg white.

## Introduction

Egg white (**EW**) is particularly valued for its high-quality protein content, which represents about 11.5% of its total composition. The major proteins in EW include ovalbumin, ovotransferrin, ovomucoid, and lysozyme, accounting for approximately 54%, 12%, 11%, and 3.5% of the total EW protein fraction, respectively (Chang et al., 2018; Réhault-Godbert et al., 2019). Each of these proteins plays a distinct biological and technological role: ovomucoid contributes to foam stability, while being also the main allergen in EW (Kavimughil et al., 2023); lysozyme is well known for its antimicrobial activity and is widely used as a natural preservative in various food products (Réhault-Godbert et al., 2019); ovotransferrin is recognized for its iron-binding capacity (Chang et al., 2018); ovalbumin, the most abundant protein in EW, provides a rich source of essential amino acids, making it highly nutritionally valuable (Chang et al., 2018). In 2023, the global market for pasteurized eggs was valued at approximately USD 82.56 billion and is projected to reach USD 201.14 billion by 2032 (Zion Market Research, 2024). This substantial growth highlights the importance of understanding the characteristics and potential applications of pasteurized egg components. Among these, pasteurized egg white (**PEW**) offers notable advantages, including a high microbiological safety and easier handling compared to EW (Pei et al., 2020). However, the high temperatures adopted during the pasteurization process can induce protein aggregation and denaturation, which may compromise the foaming, emulsifying, and gelling properties of PEW, as technological attributes of great importance for both industry and consumers (Liu et al., 2022). For this reason, pasteurization processes must be monitored and optimized to preserve overall egg quality, with particular attention to minimizing protein denaturation. On this background, several studies have demonstrated that some EW proteins (including livetins and globulins) are highly susceptible to heat-induced denaturation, whereas others (including ovotransferrin and ovalbumin) are comparatively more heat-stable (Chang et al., 2018).

To date, a variety of analytical methods have been employed to characterize EW proteins, including gel-permeation chromatography, enzyme-linked immunosorbent assay, mass spectrometry, high-pressure liquid chromatography (**HPLC**) with anion-exchange columns, and reversed-phase high-pressure liquid chromatography (**RP-HPLC**). Although RP-HPLC has been proposed as an appropriate method for detecting ovomucoid, lysozyme, and ovotransferrin, available data on repeatability, reproducibility, and linearity remain fragmented (Schäfer et al., 1999). Moreover, while previous studies have addressed the thermal stability of EW proteins, the quantification of these proteins in commercial PEW remains unexplored.

On this background, the aim of the present study was to evaluate the repeatability, reproducibility, linearity, and recovery of a RP-HPLC method for simultaneous quantification of ovomucoid, lysozyme, ovotransferrin, and ovalbumin in commercial PEW.

## Materials and methods

### Reagents and apparatus

Ultrapure water (18.2 MΩ·cm resistivity at 25°C) was obtained using an Arium Basic system (Sartorius Stedim Biotech, Varedo, Italy). Protein standards of ovomucoid, lysozyme, ovotransferrin, and ovalbumin were purchased from Sigma-Aldrich (St. Louis, MO, USA) at the highest purity level.

Chromatographic analyses were performed on a RP-HPLC apparatus (Agilent 1260 Infinity II LC system, Agilent Technologies, Santa Clara, CA), equipped with a quaternary pump (Agilent 1260 Infinity II, G7111B), a diode array detector (Agilent 1260 Infinity II, G7115A), and a refrigerated autosampler (Agilent 1260 Infinity II, G7129A).

### Experimental design and sample preparation

Ten cartons of refrigerated PEW, each representing a different commercial brand, were purchased from six supermarkets in the Veneto region (Italy). On the first day of analysis (Day 1), three aliquots were collected from each of the ten cartons and immediately subjected to RP-HPLC. The same procedure was repeated daily for four consecutive days (Day 2, Day 3, Day 4, and Day 5), resulting in a total of 15 aliquots per brand and 150 aliquots overall.

Sample preparation was adapted from Schäfer et al. (1999) and carried out by a single operator. For each sample, 250 µL of PEW was transferred into 1.5 mL microtubes, diluted with 1180 µL of ultrapure water, and sonicated for 5 min at 40 kHz (±5 %). Samples were centrifuged at 13,000 *g* for 15 min*,* and 500 µL of the resulting supernatant was diluted with acetate buffer (50 mM, pH 4.0) at a 1:3 ratio. After a second centrifugation at 13,000 *g* for 15 min, 500 µL of the resulting supernatant was mixed with 1 mL of 20% acetonitrile (**ACN**) containing 0.2% trifluoroacetic acid (**TFA**). After a third final centrifugation at 13,000 *g* for 15 min, 1 mL of the resulting supernatant was filtered through a 0.22 µm syringe filter and transferred into glass vials for RP-HPLC analysis.

### Chromatographic analysis

Chromatographic separation of ovomucoid, lysozyme, ovotransferrin, and ovalbumin was performed according to the method proposed by Schäfer et al. (1999). Analyses were conducted using a Phenomenex Jupiter C4 column (250 × 4.6 mm, 5 µm particle size), coupled with a pre-column SecurityGuard cartridge (Phenomenex KJO-4282). The mobile phases consisted of solvent A (99.9% H₂O with 0.1% TFA) and solvent B (99.9% ACN with 0.1% TFA). The injection volume was 10 μL, the column was maintained at 50°C, and detection was carried out at 215 nm with a flow rate of 0.8 mL/min. The auto-sampler kept vials at 9°C during the entire run.

The gradient profile was as follows: 10% to 17.5% B in 1 min, 17.5% to 34.8% B in 9 min, 34.8% to 43.8% B in 10 min, 43.8% to 48.2% B in 3 min, 48.2% to 50% B in 1 min, 50% to 90% B in 8 min, held at 90% B for 0.5 min, returned to 17.5% B in 0.5 min, and equilibrated for 1 min, for a total runtime of 34 min.

Data were acquired and processed using OpenLab CDS 2 software (Agilent Technologies, Santa Clara, CA). Identification of target peaks was based on retention times of standards, while quantification was achieved using a compound-specific 5-point calibration curve. All calibration curves exhibited a coefficient of determination greater than or equal to 0.99.

### Repeatability and reproducibility

Method repeatability was assessed for each of the ten commercial samples and each of the four protein fractions by calculating the relative standard deviation of concentrations measured via RP-HPLC over 3 intra-day replicates (Biswas et al., 2011). Also, method reproducibility was assessed for each of the ten commercial samples and each of the four protein fractions, but in this case, the relative standard deviation was calculated on concentrations measured over 15 replicates obtained across 5 days of analysis (Biswas et al., 2011). For clarity in data presentation and interpretation, such values were averaged over the ten commercial samples to provide mean relative standard deviation in repeatability (**RSD_r_**) and mean relative standard deviation in reproducibility (**RSD_R_**).

### Linearity and recovery

Linearity was assessed on one representative commercial PEW sample by calculating R² of the regressions between peak area and nominal concentrations of ovomucoid, lysozyme, ovotransferrin, and ovalbumin across ten serial dilutions of PEW in water (1:20, 1:30, 1:35, 1:40, 1:45, 1:50, 1:65, 1:70, 1:90, and 1:100).

Recovery was determined on one representative commercial PEW sample through spiking trials at three concentration levels, performed directly on freshly prepared PEW aliquots. Standard solutions were prepared at 10 mg/mL for ovomucoid, 5 mg/mL for lysozyme, 10 mg/mL for ovotransferrin, and 20 mg/mL for ovalbumin. Spiked samples were obtained by mixing defined volumes of PEW, ultrapure water, and protein standards to yield low, medium, and high spike levels, corresponding to final concentrations of 80, 140, and 250 µg/mL for ovomucoid; 40, 75, and 90 µg/mL for lysozyme; 75, 135, and 255 µg/mL for ovotransferrin; and 320, 415, and 480 µg/mL for ovalbumin, respectively. Recovery was expressed as the percentage ratio between the measured concentration in spiked samples and the expected concentration (Francisco and Resurreccion, 2009):

$$Recovery\;\left(\%\right)=\left(\frac{Measured\;concentration\;}{Expected\;concentration}\right)\;\times \;100$$

## Results and discussion

### Repeatability and reproducibility

Data on method repeatability expressed as average RSD_r_ are summarized in Table 1. Across the five days of analysis, ovotransferrin and lysozyme consistently showed the lowest and the highest RSD_r_, which translates to the best and the poorest repeatability, respectively. On Day 1, RSD_r_ values were 8.30% for ovomucoid, 11.40% for lysozyme, 4.71% for ovotransferrin, and 6.91% for ovalbumin. A similar pattern was observed on Day 2, although all proteins displayed slightly lower RSD_r_ values, indicating improved repeatability. Day 3, in contrast, recorded the highest variability for all proteins, which may reflect greater operator-related or instrumental fluctuations. Overall, replicates performed within the fourth day of analyses (Day 4) showed the best repeatability, with the lowest RSD_r_ values: 4.30% for ovomucoid, 9.74% for lysozyme, 4.26% for ovotransferrin, and 5.12% for ovalbumin. In our study, ovotransferrin showed the lowest variability; a possible explanation could be the differences in the relative abundance of ovotransferrin, as a higher concentration of this protein may enhance signal stability and reduce measurement variability. Conversely, lysozyme showed the highest RSD_r_. This apparent inconsistency likely reflects, to some extent, the relatively low abundance of lysozyme in PEW, which hampers accurate quantification and increases data variability.

**Table 1:** Average relative standard deviation in repeatability (RSD_r_) and average relative standard deviation in reproducibility (RSD_R_) for ovomucoid, lysozyme, ovotransferrin, and ovalbumin.

Protein	RSD_r_ (%)					RSD_R_ (%)
	Day 1	Day 2	Day 3	Day 4	Day 5	
Ovomucoid	8.30	5.96	9.68	4.30	6.87	5.75
Lysozyme	11.40	10.78	14.10	9.74	12.19	9.53
Ovotransferrin	4.71	4.51	7.32	4.26	4.72	4.81
Ovalbumin	6.91	5.42	9.91	5.12	7.16	6.48

Average RSD_R_ values, indicative of method reproducibility, are reported in Table 1. The lowest RSD_R_ (i.e., the greatest reproducibility) was observed for ovotransferrin (4.81%), followed by ovomucoid (5.75%), ovalbumin (6.48%), and lysozyme (9.53%). These results reinforce the consistency of ovotransferrin measurements and highlight the relative instability of lysozyme quantification across multiple days. Notably, RSD_R_ values obtained for ovomucoid are consistent with those reported by Kavimughil et al. (2023), who observed CV ranging between 1.8% and 9.7% when analyzing standard solutions of ovomucoid in the 5-100 µg/mL range. Overall, RSD_R_ was generally lower than RSD_r,_ confirming the method’s robustness and suitability for routine application in quality control or process optimization settings.

To the best of our knowledge, no previous studies have evaluated repeatability and reproducibility of the chromatographic method for protein quantification in PEW. Previous works have mainly relied on analytical performances of other alternative techniques such as ELISA, SDS-PAGE, or mass spectrometry (Francisco and Resurreccion, 2009; Chang et al., 2018). Although these approaches have been successfully used for EW protein identification or semi-quantitative analysis, they present several limitations for accurate and simultaneous quantification of multiple proteins in complex food matrices. In particular, ELISA and SDS-PAGE require extensive sample preparation and are typically restricted to single-target or qualitative assessments. On the other hand, mass spectrometry, despite being characterized by high sensitivity and specificity, involves complex and time-consuming workflows that limit its routine applicability. In contrast, the present RP-HPLC method allows the efficient characterization of the major proteins in PEW, without requiring additional purification or concentration steps, while providing adequate repeatability and reproducibility. Moreover, most previous studies have relied on purified or lyophilized albumen (Chang et al., 2018), meaning EW that has been isolated and further processed to remove other components, thus providing a simplified and controlled composition. In contrast, the use of PEW in this study represents a more challenging analytical substrate because of potential interfering components such as salts, sugars, and residual lipids, which can affect method repeatability and reproducibility. Importantly, the experimental design adopted in the present study was conceived to validate the analytical method under real matrix conditions, ensuring its applicability to actual food samples rather than in controlled or simplified laboratory models.

### Recovery and linearity

Method recovery for ovomucoid, lysozyme, ovotransferrin, and ovalbumin in commercial PEW is in Table 2. Recovery rates ranged from 90.67% (for lysozyme at the medium spiking level) to 114.10% (for ovomucoid at the medium spiking level). While recoveries below 100% indicate underestimation of the target protein, recoveries above 100% indicate overestimation. The largest deviations from the ideal value of 100% were observed for ovomucoid, while the smallest were obtained for ovotransferrin and ovalbumin. Recoveries for lysozyme were consistently below 100% which may be related to its relatively low concentration in PEW, as well as its lower molecular weight and higher hydrophilicity, potentially affecting adsorption during filtration or chromatographic separation (Kavimughil et al., 2023). Recovery values close to 100% were obtained for ovotransferrin and ovalbumin, confirming the efficiency of the method even for proteins differing in structure and polarity. Notably, Itoh et al. (1991) reported a recovery rate of approximately 86% for ovalbumin during rapid separation, indicating that the conditions used in the present study improved analytical performance. Overall, recovery rates observed in the present study are in the range of those expected for protein quantification performed through RP-HPLC (80-120%; Francisco and Resurreccion, 2009), indicating that the sample preparation and chromatographic separation steps ensured efficient extraction and accurate quantification of the target compounds.

**Table 2:** Recovery^1^, linearity^2^, and descriptive statistics (calculated on 10 samples analyzed 15 times each) for ovomucoid, lysozyme, ovotransferrin, and ovalbumin in commercial pasteurized egg white samples.

Protein	Recovery (%)			Linearity	Descriptive statistics (mg/mL; n = 150)	
	Low Spike	Medium Spike	High Spike	R^2^	Mean	Standard deviation
Ovomucoid	112.90	114.10	109.37	0.990	15.23	1.02
Lysozyme	92.63	90.67	93.38	0.994	2.23	0.23
Ovotransferrin	101.72	101.00	98.65	0.993	13.50	0.74
Ovalbumin	100.44	100.50	97.77	0.990	71.80	5.48

1 Low spike level: 1180 µL of the standard mix, and 250 µL of PEW, resulting in final concentrations of 80 µg/mL for ovomucoid, 40 µg/mL for lysozyme, 75 µg/mL for ovotransferrin, and 320 µg/mL for ovalbumin; medium spike level: 800 µL of the standard mix, 380 µL of ultrapure water, and 250 µL of PEW, resulting in final concentrations of 140 µg/mL for ovomucoid, 75 µg/mL for lysozyme, 135 µg/mL for ovotransferrin, and 415 µg/mL for ovalbumin; high spike level: 500 µL of the standard mix, 680 µL of ultrapure water, and 250 µL of PEW, resulting in final concentrations of 250 µg/mL for ovomucoid, 90 µg/mL for lysozyme, 255 µg/mL for ovotransferrin, and 480 µg/mL for ovalbumin.

2 Expressed as the coefficient of determination (R²) obtained from the regression of peak area versus nominal protein concentration across ten serial aqueous dilutions of the sample.

Equations obtained from the linearity tests expressed as y = mx + b (where y is the chromatographic peak area, x the analyte concentration, m the slope, and b the y-intercept) are as follows: y=779.64x−4.0364 (for ovomucoid), y=184.03x−1.8465 (for lysozyme), y=588.63x−1.5599 (for ovotransferrin), and y=3672.1x−23.014(for ovalbumin). The coefficient of determination (R²) obtained from these regressions quantifies the goodness of fit between experimental data and the model, reflecting the consistency and reliability of the analytical response across the tested concentration range (Table 2). In this study, all R² values were ≥ 0.99, namely 0.990 for ovomucoid, 0.994 for lysozyme, 0.993 for ovotransferrin, and 0.990 for ovalbumin, indicating excellent linearity and a highly reliable quantitative correlation between protein concentration and instrument signal. Such strong correlations confirm that the analytical method provides stable and proportional responses within the studied range.

### Method generalizability and variability of protein content in pasteurized egg white

The analysis revealed mean concentrations of 15.23, 2.23, 13.50, and 71.80 mg/mL for ovomucoid, lysozyme, ovotransferrin, and ovalbumin, respectively (Table 2). These results are in agreement with those reported for non-pasteurized EW (Chang et al., 2018; Réhault-Godbert et al., 2019), indicating that the pasteurization process applied to commercial samples preserved the overall protein composition of the matrix.

Minor modifications to the method of Schäfer et al. (1999) were introduced to improve resolution and analytical consistency. First, a C4 reversed-phase column was employed instead of the original C18, as it provides better selectivity for large and moderately hydrophobic proteins such as ovalbumin and ovotransferrin. Additionally, the elution gradient was optimized to achieve sharper and more symmetrical peaks, while maintaining an acceptable analysis time. Moreover, sample temperature was controlled throughout the analysis to minimize protein precipitation and variability in retention times. These adjustments resulted in improved peak separation and lower within-day and between-day variability, confirming that RP-HPLC can be effectively adopted for the quantitative analysis of major proteins in PEW, providing a reliable tool for both scientific research and industrial applications.

Overall, the method exhibited satisfactory repeatability and reproducibility across all target compounds, with ovotransferrin and ovalbumin showing particularly consistent performances. From a technological standpoint, the good linearity and high recovery observed for all proteins are particularly relevant, as these molecules contribute significantly to the functional and technological properties of EW. The ability to accurately quantify these proteins thus provides a valuable analytical tool for monitoring processing effects, such as pasteurization or storage, on the functional quality of EW. Results of the present study confirm the suitability of RP-HPLC for routine protein analysis in complex food matrices and highlight its potential to support both research and industrial applications related to the quality control and functional characterization of commercial EW products.

## CRediT authorship contribution statement

**Ingrid Sousa:** Writing – original draft, Visualization, Software, Methodology, Formal analysis, Data curation. **Elena Visentin:** Writing – original draft, Visualization, Software, Methodology, Formal analysis, Data curation. **Silvia Sabbadin:** Writing – review & editing, Visualization, Software, Methodology, Formal analysis. **Marta Pozza:** Writing – review & editing, Visualization, Software, Methodology. **Marco Birolo:** Writing – review & editing, Visualization, Validation, Data curation, Conceptualization. **Massimo De Marchi:** Writing – review & editing, Supervision, Resources, Project administration, Funding acquisition, Conceptualization. **Giovanni Niero:** Writing – review & editing, Writing – original draft, Visualization, Validation, Supervision, Investigation, Data curation, Conceptualization.

## Disclosures

The authors declare that they have no known competing financial interests or personal relationships that could have appeared to influence the work reported in this paper.
